# Sticky Gecko Feet: The Role of Temperature and Humidity

**DOI:** 10.1371/journal.pone.0002192

**Published:** 2008-05-14

**Authors:** Peter H. Niewiarowski, Stephanie Lopez, Liehui Ge, Emily Hagan, Ali Dhinojwala

**Affiliations:** 1 Department of Biology, Program in Integrated Bioscience, University of Akron, Akron, Ohio, United States of America; 2 Department of Polymer Science, University of Akron, Akron, Ohio, United States of America; Center for Genomic Regulation, Spain

## Abstract

Gecko adhesion is expected to be temperature insensitive over the range of temperatures typically experienced by geckos. Previous work is limited and equivocal on whether this expectation holds. We tested the temperature dependence of adhesion in Tokay and Day geckos and found that clinging ability at 12°C was nearly double the clinging ability at 32°C. However, rather than confirming a simple temperature effect, our data reveal a complex interaction between temperature and humidity that can drive differences in adhesion by as much as two-fold. Our findings have important implications for inferences about the mechanisms underlying the exceptional clinging capabilities of geckos, including whether performance of free-ranging animals is based solely on a dry adhesive model. An understanding of the relative contributions of van der Waals interactions and how humidity and temperature variation affects clinging capacities will be required to test hypotheses about the evolution of gecko toepads and is relevant to the design and manufacture of synthetic mimics.

## Introduction

An average sized (40–50 g) adult Tokay gecko (*Gekko gecko*) can generate the equivalent of more than 100 times its own body weight in clinging force (∼40 N) using its dry adhesive toepads [1]. Although quantitative measurements of maximal clinging capacities, and characterization of the underlying microscopic functional morphology is a relatively recent accomplishment [2], researchers have been proposing hypotheses and debating mechanisms responsible for such remarkable clinging abilities for over 100 years [2]. Despite a burgeoning literature on gecko adhesion and the obvious applications to evolutionary biologists and materials scientists alike [3], there are still significant gaps in our understanding of fundamental aspects of gecko adhesion at multiple scales of analysis. For example, the functional and evolutionary significance of variation in toepad and setal morphology was explicitly raised nearly 50 years ago [4], [5], however, studies have only just begun to address such relationships [6], [7]. Similarly, locomotor capacities of ectotherms like lizards have been subject to extensive scrutiny focusing on the temperature sensitivity of performance [8], but only recently has the study of the temperature sensitivity of gecko clinging ability joined this literature [9], [10].

Studies of gecko adhesion at the setal and spatulae level, suggest that intermolecular interactions (van der Waals forces) are responsible for adhesion. If clinging in geckos is only based on van der Waals forces [11], clinging capacity should be temperature insensitive over the range of body temperatures typically experienced by geckos [10]. Indeed, that a key functional capacity of locomotion in geckos may be free of typical thermal biophysical constraints experienced by ectotherms in thermally heterogeneous environments, suggests that thermal independence of adhesion may have driven the evolution of the gecko adhesive system [10]. Temperature sensitivity of clinging capacity has only been measured in two species (*Phelsum dubia* and *Gekko gecko*; [9], [10]). Moreover, contrasting results between these two studies are difficult to interpret because different methodologies were employed, leaving the question of the temperature sensitivity of clinging capacity unanswered. In an effort to eliminate the potential contribution of divergent methods used to evaluate the temperature sensitivity of clinging performance, we describe a series of experiments that estimated maximal clinging ability in *P. dubia* and *G. gecko* over a range of biologically meaningful temperatures, using a single protocol. During the conduct of our experiments, it became clear that interpreting temperature sensitivity of clinging ability requires knowledge and control of relative humidity, a potential source of variation noted but discounted in previous studies [9]. However, variation in humidity has recently been implicated as a source of variation in adhesive forces generated at the nanoscale between single spatulae and various substrates [12].

## Results

### Temperature effects

**Figure 1:**
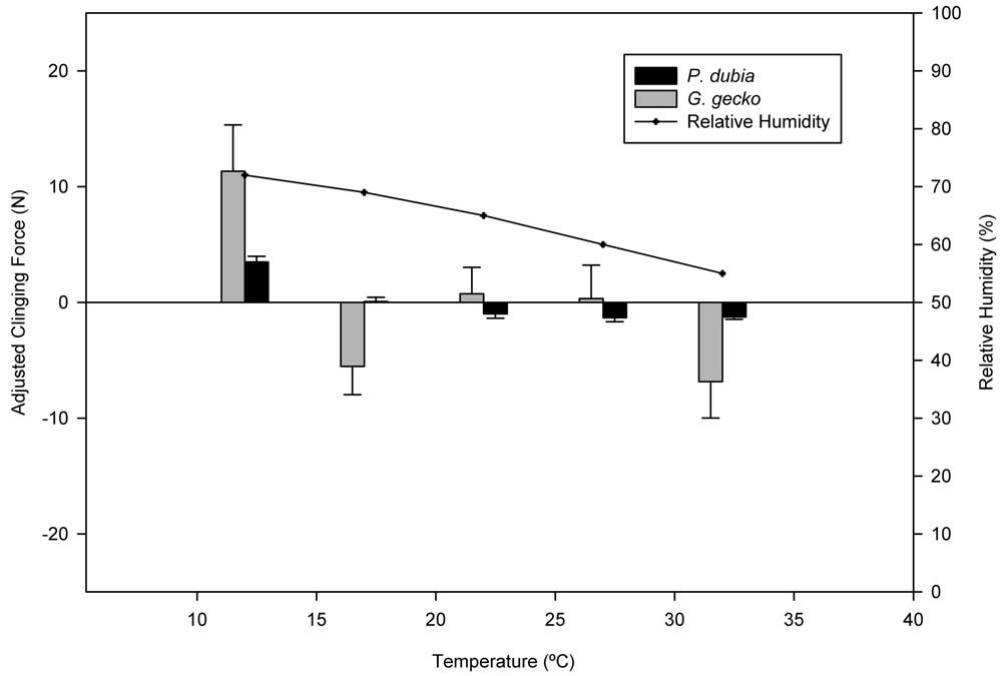
Experimental trials at variable temperatures without controlling for humidity. Relationship between temperature and body-size corrected adhesion (clinging force [N]; left axis, bars) or relative humidity (%; right axis, solid line).

Tokay geckos were larger (58.14±6.8 g) and had greater total toepad areas (4.74±0.98 cm^2^) than Day geckos (5.58±0.32 g and 0.73±0.06 cm^2^, respectively; Table 1). Tokays were also able to generate higher maximal total clinging forces (31.22±4.39 N and 5.53±0.51N, respectively, *F_1,15_* 29.9, *P*<0.0001), but maximal force per unit toepad area was similar between species (7.83±1.72 N/cm^2^ and 6.34±1.62 N/cm^2^, Day and Tokay respectively; *F_1,15_* = 1.6, *P* = 0.23). Adjusted maximal clinging force was significantly different across temperatures (*F_4,56_* = 8.09, *P*<0.0001), and there was a significant difference between species in the effect of temperature on clinging force (species*temperature *F_4,56_* = 2.91, *P* = 0.03) leading us to analyze the effect of temperature separately for each species. Although the effect of temperature was significant for both Day and Tokay geckos (*F_4,28_* = 28.49, *P*<0.0001 and *F_4,28_* = 5.05, *P* = 0.003, respectively), the trend for an increase in clinging ability with decreasing temperature appeared stronger for Day geckos (Fig. 1). For example, the clinging ability of Tokay geckos at the intermediate temperature (22°C) was close to but not quite significantly different from clinging ability at the lowest temperature (12°C; matched pairs t-Test, *t* = 2.23, *P* = 0.056) but clinging force at 12°C was significantly higher than at 22°C for Day geckos (12°C; matched pairs t-Test, *t* = 10.78, *P*<0.0001).

**Table 1:** Sex, size, and toe pad area of geckos used in experimental trials. Toe pad area is an estimate of maximal area of scansors visible on scans.

Individual	Sex	Weight (g)	Toe Pad Area (cm^2^)
day 1	Female	5.30	0.64
day 2	Female	5.58	0.72
day 3	Male	4.96	0.66
day 5	Male	4.72	0.65
day 6	Female	5.48	0.73
day 7	Female	7.26	0.84
day 8	Female	5.50	0.72
day 9	Female	6.90	0.79
day 12	Male	6.2	1.0
day 14	Male	3.9	0.591
tokay 1	Male	68.80	4.65
tokay 2	Male	59.34	4.95
tokay 3	Male	59.14	4.87
tokay 4	Female	73.24	5.46
tokay 5	Male	54.30	3.93
tokay 6	Male	78.16	5.31
tokay 7	Female	69.22	4.49
tokay 8	Male	79.90	5.27
tokay 9	Female	73.30	4.91
tokay 10	Male	47.8	4.9
tokay 11	Female	42.8	4.06
tokay 12	Female	44.9	4.32
tokay 14	Female	46.4	4.47
tokay 17	Male	43.8	4.57
tokay 18	Male	50.0	5.55
tokay 19	Female	39.2	4.15

### Humidity effects

**Figure 2:**
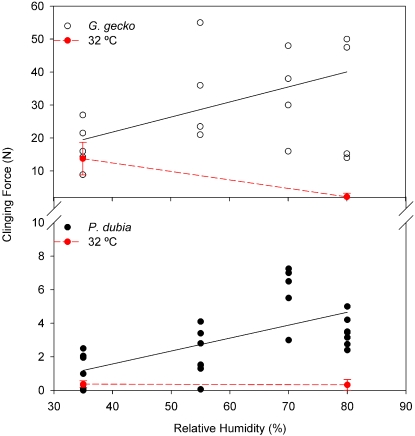
Adhesion at constant temperature of 12°C and variable relative humidity (RH). Adhesion increased significantly with RH, but slopes were not significantly different. Red lines and symbols show results of 35% and 80% trials at 32°C designed to test for an interaction of temperature and humidity; error bars represent ±2 SE. See text for details (materials and methods).

At 12°C, clinging ability increased significantly with humidity (Fig. 2; *F_3,27_* = 4.61, *P* = 0.01), such that forces measured at the highest humidity (80%) were nearly twice as high as the forces observed at the lowest humidity (35%). Although there was a trend suggesting the effect of humidity was stronger for Day geckos, there was no significant difference between species in the rate of change in clinging force with humidity (species*rh *F_3,27_* = 2.28, *P* = 0.1). In order to determine if the humidity effect observed at 12°C varied with temperature, we did one last set of trials measuring clinging ability at 32°C at 35% and 80% RH. Surprisingly, at 32°C, clinging ability did not vary in response to humidity as it did at 12°C (Fig. 2.). Instead, the clinging ability of Tokay geckos was significantly higher at 35% RH compared to 80% RH (13.75±3.0 N vs. 2.48±2.0 N; *F_1,15_* = 38.9, *P*<0.0001), while the clinging ability of Day geckos did not vary significantly between 35% and 80% RH (0.375±0.2 N vs. 0.327±0.36 N; *F_1,15_* = 0.05, *P* = 0.8261). Moreover, at 32°C clinging ability was among the lowest measured in any set of trials.

## Discussion

A preponderance of evidence suggests that geckos stick to substrates as a consequence of the formation of a large number of intimate setal-substrate contacts engaging van der Waals attraction. One prediction about performance that we can derive from this mechanism is that adhesion should be temperature insensitive over a biologically meaningful range [1]. However, previous work, though limited in scope, is equivocal on this prediction [9], [10]. Admittedly, such a simple prediction ignores important details of adhesion at the whole organism scale. For example, adhesion may involve more than just the sum of the setal-substrate interactions; the contributions of elaborate subcutaneous vascular, muscular and skeletal elements in adhesion and release is currently completely unexplored [2], [10]. Presumably, these components would be subject to thermal dependencies typical of ectotherms [13]. Our experiment measured adhesion at different temperatures using a single protocol with two species that have been previously studied (*G. gecko* and *P. dubia*). Although we found evidence of a strong effect of temperature, the direction of the effect was counterintuitive given the thermal biology of geckos and it violated the prediction given by van der Waals interactions presumed responsible for adhesion. Consequently, we wanted to examine whether other factors (e.g., humidity) could explain the variation in clinging performance we observed. We found evidence, unexpectedly, that humidity is likely an important determinant of clinging force in the geckos we studied. Below we explore implications of our results for understanding both the factors affecting adhesion in geckos, as well as for inferring mechanisms that underlie such performance.

**Figure 3:**
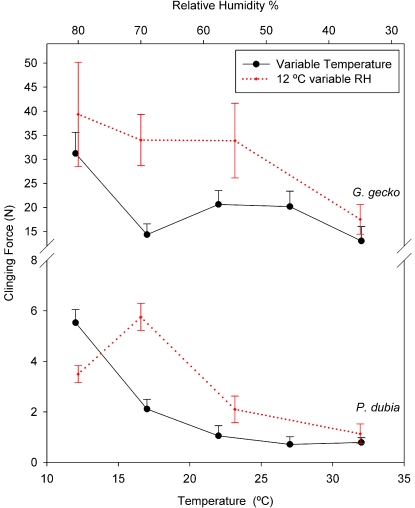
Adhesion during variable temperature, uncontrolled humidity trials (black solid lines), and variable relative humidity constant temperature trials (12°C; red-dotted lines) showing convergence of maximal clinging force with increasing temperature and decreasing humidity. Error bars are ±2 SEM.

Restricting attention just to temperature effects (Table 2 and Fig. 1), leads to the conclusion that adhesion is highly temperature sensitive, in both species, with greatest adhesion observed at the lowest test temperature. However, RH varied by approximately 15% between the two temperature extremes (12°C and 32°C; Fig. 1). It is important to note that relative humidity was not controlled in previous studies that examined temperature effects on adhesion in these two species, but differences in protocol are intriguing. Bergmann and Irschick [10], who found no evidence of a temperature effect on clinging ability of *P. dubia* heated or cooled geckos in an environmental chamber and then performed clinging tests at room temperature and ambient humidity conditions of their laboratory. Alternatively, Losos [9], performed clinging tests on *G. gecko* inside a walk-in environmental chamber set to one of 9 different temperatures. As in our design, relative humidity in the latter study presumably varied with temperature, and a strong effect of temperature on clinging ability was demonstrated. Notably, adhesion in the Losos study [9] was maximal at intermediate temperatures (which corresponded to the highest relative humidity). Consideration of both our temperature and 12°C humidity trials (Fig. 3), in light of the two studies described above, suggests that clinging ability is sensitive to variation in humidity, not temperature. Unfortunately, the humidity response does not appear to be simple. Indeed, the response to variation in humidity at 12°C is not constant but apparently itself is affected by temperature (Fig. 2). In other words, clinging ability by geckos on a smooth hydrophilic surface like glass appears to be sensitive to both temperature and humidity.

**Table 2:** Average maximal adjusted adhesive force (N) by species and temperature. Errors are ±1 SEM.

	Temperature (°C)				
Species	12	17	22	27	32
day	5.53±0.51	2.11±0.38	1.05±0.40	0.71±0.30	0.79±0.19
tokay	31.22±4.39	14.37±2.20	20.64±2.87	20.19±3.18	13.05±2.96

Interestingly, the effect of humidity on gecko adhesion has never been formally investigated at the whole animal scale that we are aware. However, two studies focusing on elucidating the mechanism underlying the setal-substrate interaction have demonstrated that adhesive forces between a single spatula and substrate *is* humidity dependent (at room temperature) [12], [14]. Scaling of the effect of humidity at the nano-scale shows an approximately 1.3 fold increase in adhesion between 35% and 80% RH [12], or a 3-fold difference between 0 and 70% RH [14]. Our study shows that at the whole animal scale the effect of humidity is quite complex. We have observed an approximate doubling of adhesion over 35 to 80% RH at 12°C. On the other hand, the adhesion forces are relatively insensitive to humidity at 35°C. We note, however, there is now an important gap in experiments at the setal scale: humidity manipulations have only been done at a single temperature.

The strong influence of humidity on the adhesive forces at a single setal and whole animal level also suggests that capillarity forces may play an important role in adhesion. A strong effect of humidity on adhesion forces has also been observed using atomic force microscopy for hydrophilic tips in contact with hydrophilic surfaces. Alternatively, when one of the materials is hydrophobic, adhesion is humidity independent [15]. Our study raises several paradoxes that cannot be resolved with existing data or theory. First, the humidity dependent adhesion forces at 12°C suggest that setae must be hydrophilic, despite evidence that water droplets do not wet gecko feet and roll off easily upon tilting. Second, it is not clear why humidity has a strong influence on the adhesion forces only at low temperatures. A capillary model predicts that the adhesion forces at the same humidity should be proportional to temperature in units of Kelvin [16]. Therefore, the adhesion forces at 35°C should be only 8% higher than the adhesion forces at 12°C. These predictions are much smaller than experimental observations. In addition, the capillary model predicts the adhesion forces at 35°C should show similar dependence on humidity. Both the van der Waals and capillary forces fail to explain the shear adhesion data at the whole animal level. Resolution of this paradox will likely require examination of the particular way in which water interacts with substrate and seta at the nanoscale level. In addition, the adhesion force measurements at a single setal scale are needed at variable temperatures before we can eliminate the possibility of thermal biophysical constraints influencing the adhesion.

Irrespective of the mechanism, effects of humidity on otherwise “dry adhesive” biological systems may not be limited to gecko toe pads. For example, members of the spider infraorder Araneomorphae produce a derived type of silk which adheres to surfaces with greater force as humidity increases [17], [18]. It has been hypothesized that regularly spaced nodes in the silk strands are richer in hydrophilic polar or charged amino acids, which under humid conditions should promote hydroscopic interactions between the nodes and substrates [17]. It is unclear whether similar mechanisms might underlie the humidity response of gecko toe pads. However, efforts to characterize the protein structure of setae have revealed that setae have a complex structure of heterogeneous α- and β-keratins [19]–[21]. Furthermore, there is evidence that the lateral regions of the β-keratins sequenced in gecko setae are relatively rich in hydrophilic and polar amino acids that could modify adhesive interactions under moderate humidity conditions [22], perhaps in a way analogous to the increased stickiness of nodes in silk of some spiders in the Araneomorphae.

In summary, our findings are partially consistent with those of studies examining the effects of humidity on adhesion in individual setae [12], [14]. Adhesion increases similarly with humidity at both the nano and whole organism scale, suggesting that van der Waals forces may not provide a complete description of the mechanics responsible for clinging in geckos. Implications are potentially broad-reaching, both for biological hypotheses about the evolution of toe pads, as well as for bioengineers interested in biologically-inspired design of synthetic adhesives.

## Materials and Methods

We obtained 10 *Phelsuma dubia* (Dull Day Gecko) and 16 *Gekko gecko* (Tokay Gecko) from California Zoological Supply. *Phelsuma. dubia* is a diurnal gecko found in Madagascar. Our specimens weighed between 4.7–7.3 g. *Gekko gecko*, is a nocturnal gecko found in Southeast Asia. Our specimens weighed between 54–80 g. All specimens were housed in individual 10 gallon glass tanks with paper towel substrate, maintained within a dedicated animal research facility at the University of Akron. Each tank was misted with water as well as provided with a fresh bowl of water daily. *G. gecko* were fed vitamin dusted crickets 5 times a week whereas the *P. dubia* were fed vitamin dusted crickets 3 times a week and the other 2 days they received a fruit supplement. The room was maintained at 25±1°C and set to a 12 hour photoperiod with UVA/UVB full spectrum lights. Heating tape along the underside of each tank allowed geckos to thermoregulate within the range of body temperatures typical for free-ranging geckos (∼25–35°C).

### Temperature trials

**Figure 4:**
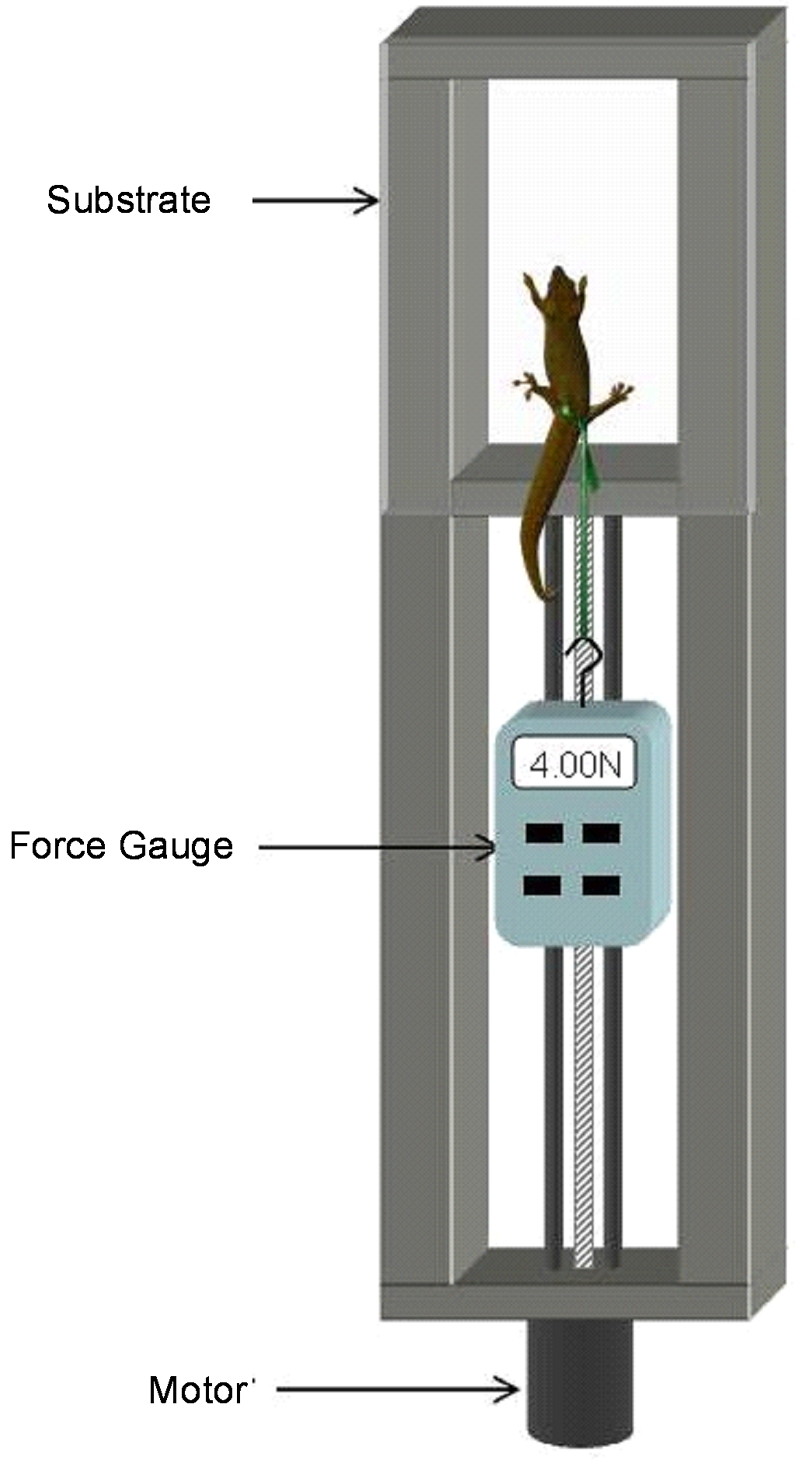
Force measuring apparatus. Geckos could be pulled at a constant rate selectable over a wide range of values. All pulls were accomplished with the substrate in a vertical orientation. Maximum force was the highest value recorded between the start of a pull and the point at which all four feet began to slide on the substrate.

We used a walk in environmental chamber maintained to within±1°C of five randomly presented experimental temperatures, 12, 17, 22, 27, and 32°C. Each set of trials was initiated approximately two hours into the species-specific diel-cycle (9am for *P. dubia* and 9pm for *G. gecko*). Geckos were placed inside the environmental chamber from 1-2 hours prior to all experimental procedures to allow body temperature equilibration. No more than a single temperature was tested on any given day. We used a specially designed apparatus (Fig. 4) to hold a clinging substrate (glass in our experiment) and a motor driven force gauge (Shimpo FGV-10X) which moved relative to the substrate. Digital output from the force gauge was collected by a LabVIEW® program allowing us to save traces of each adhesion trial. For all trials, we report values with the substrate in the vertical position. Each gecko was fitted with specially designed harnesses positioned around the pelvis and allowing unimpeded movement of legs and minimizing abrasion to the gecko's skin. At the start of each test, the harness was attached to the force gauge which was subsequently driven downward by the motor. Geckos were introduced to the substrate and induced to take step with each foot, providing a standardized posture with all four feet and footpads fully in contact with the substrate. Each individual was then pulled parallel to the surface in a smooth motion until all four feet began to slide indicating the end of the test (referred to hereafter as a ‘pull’). Geckos were pulled three times in succession to estimate maximum clinging force. Between each gecko, the glass was cleaned with ethanol and dried before the next set of pulls.

### Humidity trials

Subsequent to completing all the temperature trials, we became concerned that variation in the relative humidity of the environmental chamber at different temperatures might be a contributing factor to variation in estimates of maximum clinging ability. Consequently, we initiated a second set of trials to estimate the effect of humidity at the temperature where recorded clinging forces were maximal for both species (12°C). Humidity trials followed the protocol described above for temperature trials, except that temperature was maintained constant but relative humidity was maintained at one of four levels, presented in a random order (30, 55, 70, 80%). Humidity trials at 12°C were followed by two trials at 32°C (at 35 and 80% RH) to test whether temperature and humidity interact.

At the completion of every trial, geckos were weighed to the nearest 0.1 g. Before every trial and after the very last trial, the feet of each gecko were scanned with a flat bed scanner so we could estimate the total adhesive area of the toe pads.

### Statistical analyses

Our analyses include data from several separate experiments, each with slightly different sample sizes and overall designs. Whenever an analysis involved data from geckos exposed to all combinations of experiment factors (i.e., humidity and temperature), we used a univariate repeated measures ANOVA to test for the statistical significance of effects in the model (assumption of sphericity was never violated). Clinging force is reported in two forms, raw and “adjusted,” facilitating comparisons using both standardized and raw values. Adjusted clinging forces are the residuals from a linear regression of raw clinging force on toe pad area, providing a measure of clinging force that accounts for variation among individual lizards in toe pad size. Throughout the manuscript, whenever clinging forces are presented as ‘adjusted’ they are standardized as described above; values without the ‘adjusted’ descriptor represent the raw values. Finally, when means are reported in the text or tables they are accompanied by their respective standard errors (± 1 SEM), unless otherwise noted.

## References

[1] Autumn K (2006). How gecko toes stick-The powerful, fantastic adhesive used by geckos is made of nanoscale hairs that engage tiny forces, inspiring envy among human imitators.. American Scientist.

[2] Russell AP (2002). Integrative functional morphology of the gekkotan adhesive system (Reptilia : Gekkota).. Integrative and Comparative Biology.

[3] Pianka ER, Sweet SS (2005). Integrative biology of sticky feet in geckos.. Bioessays.

[4] Maderson PFA (1970). Lizard hands and lizard glands: Models for evolutionary study.. Forma et Functio.

[5] Williams EE, Peterson JA (1982). Convergent and Alternative Designs in the Digital Adhesive Pads of Scincid Lizards.. Science.

[6] Irschick DJ, Austin CC, Petren K, Fisher RN, Losos JB (1996). A comparative analysis of clinging ability among pad-bearing lizards.. Biological Journal of the Linnean Society.

[7] Irschick DJ, Herrel A, Vanhooydonck B (2006). Whole-organism studies of adhesion in pad-bearing lizards: creative evolutionary solutions to functional problems.. Journal of Comparative Physiology A-Neuroethology Sensory Neural and Behavioral Physiology.

[8] Huey RB, Niewiarowski PH, Kaufmann J, Herron JC (1989). Thermal Biology of Nocturnal Ectotherms-Is Sprint Performance of Geckos Maximal at Low Body Temperatures.. Physiological Zoology.

[9] Losos (1990). Thermal sensitivity of sprinting and clinging performance in the tokay gecko (*Gekko gecko*).. Asiatic Herpetological Research.

[10] Bergmann PJ, Irschick DJ (2005). Effects of temperature on maximum clinging ability in a diurnal gecko: Evidence for a passive clinging mechanism?. Journal of Experimental Zoology Part A-Comparative Experimental Biology.

[11] Autumn K, Peattie AM (2002). Mechanisms of adhesion in geckos.. Integrative and Comparative Biology.

[12] Huber G, Mantz H, Spolenak R, Mecke K, Jacobs K (2005). Evidence for capillarity contributions to gecko adhesion from single spatula nanomechanical measurements.. Proceedings of the National Academy of Sciences of the United States of America.

[13] Angilletta MJ, Niewiarowski PH, Navas CA (2002). The evolution of thermal physiology in ectotherms.. Journal of Thermal Biology.

[14] Sun WX, Neuzil P, Kustandi TS, Oh S, Samper VD (2005). The nature of the gecko lizard adhesive force.. Biophysical Journal.

[15] Qian LM, Tian F, Xiao XD (2003). Tribological properties of self-assembled monolayers and their substrates under various humid environments.. Tribology Letters.

[16] Kim TW, Bhushan B (2008). The adhesion model considering capillarity for gecko attachment system.. J R Soc Interface.

[17] Hawthorn AC, Opell BD (2003). van der Waals and hygroscopic forces of adhesion generated by spider capture threads.. Journal of Experimental Biology.

[18] Hawthorn AC, Opell BD (2002). Evolution of adhesive mechanisms in cribellar spider prey capture thread: evidence for van der Waals and hygroscopic forces.. Biological Journal of the Linnean Society.

[19] Alibardi L (2003). Ultrastructural autoradiographic and immunocytochemical analysis of setae formation and keratinization in the digital pads of the gecko *Hemidactylus turcicus* (Gekkonidae, Reptilia).. Tissue & Cell.

[20] Alibardi L, Toni M (2005). Distribution and characterization of proteins associated with cornification in the epidermis of gecko lizard.. Tissue & Cell.

[21] Alibardi L, Toni M, Valle LD (2007). Expression of beta-keratin mRNAs and proline uptake in epidermal cells of growing scales and pad lamellae of gecko lizards.. Journal of Anatomy.

[22] Toni M, Valle LD, Alibardi L (2007). The epidermis of scales in gecko lizards contains multiple forms of beta-keratins including basic glycine-proline-serine-rich proteins.. Journal of Proteome Research.

